# One Is Not Enough: Understanding and Modeling Polysubstance Use

**DOI:** 10.3389/fnins.2020.00569

**Published:** 2020-06-16

**Authors:** Elizabeth A. Crummy, Timothy J. O’Neal, Britahny M. Baskin, Susan M. Ferguson

**Affiliations:** ^1^Graduate Program in Neuroscience, University of Washington, Seattle, WA, United States; ^2^Center for Neurobiology of Addiction, Pain, and Emotion, University of Washington, Seattle, WA, United States; ^3^Department of Psychiatry and Behavioral Sciences, University of Washington, Seattle, WA, United States; ^4^Center for Integrative Brain Research, Seattle Children’s Research Institute, Seattle, WA, United States; ^5^Alcohol and Drug Abuse Institute, University of Washington, Seattle, WA, United States

**Keywords:** polydrug, substance use, addiction, reward circuitry, preclinical models, neuronal signaling and behavior, review, neurobiology of addiction

## Abstract

Substance use disorder (SUD) is a chronic, relapsing disease with a highly multifaceted pathology that includes (but is not limited to) sensitivity to drug-associated cues, negative affect, and motivation to maintain drug consumption. SUDs are highly prevalent, with 35 million people meeting criteria for SUD. While drug use and addiction are highly studied, most investigations of SUDs examine drug use in isolation, rather than in the more prevalent context of comorbid substance histories. Indeed, 11.3% of individuals diagnosed with a SUD have concurrent alcohol and illicit drug use disorders. Furthermore, having a SUD with one substance increases susceptibility to developing dependence on additional substances. For example, the increased risk of developing heroin dependence is twofold for alcohol misusers, threefold for cannabis users, 15-fold for cocaine users, and 40-fold for prescription misusers. Given the prevalence and risk associated with polysubstance use and current public health crises, examining these disorders through the lens of co-use is essential for translatability and improved treatment efficacy. The escalating economic and social costs and continued rise in drug use has spurred interest in developing preclinical models that effectively model this phenomenon. Here, we review the current state of the field in understanding the behavioral and neural circuitry in the context of co-use with common pairings of alcohol, nicotine, cannabis, and other addictive substances. Moreover, we outline key considerations when developing polysubstance models, including challenges to developing preclinical models to provide insights and improve treatment outcomes.

## Introduction

**FIGURE 1:**
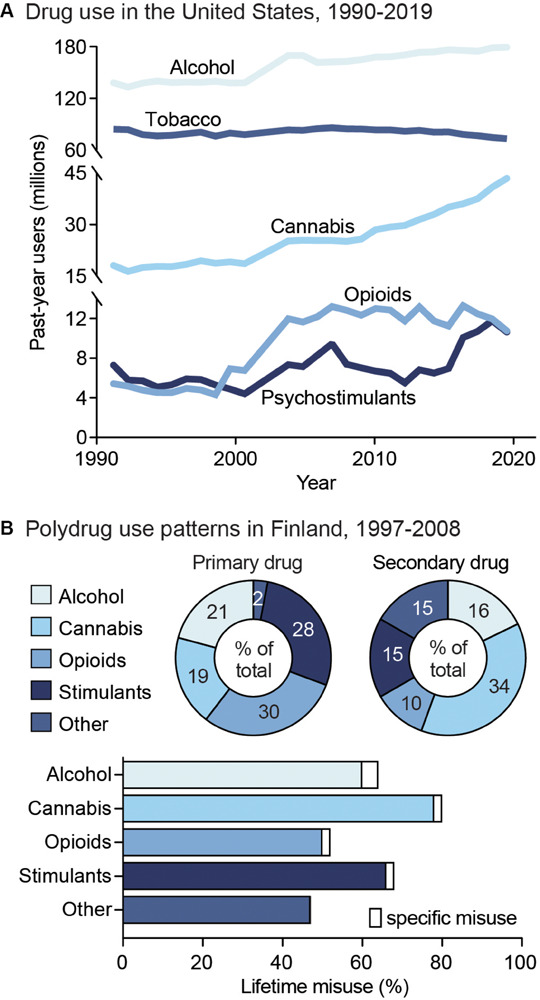
Public health trends in drug use. **(A)** Drug use in the United States from 1990 to 2019. Data from the National Household Survey on Drug Abuse (Substance Abuse and Mental Health Services Administration, 1993; Substance Abuse and Mental Health Services Administration, 1995; Substance Abuse and Mental Health Services Administration, 1997; Substance Abuse and Mental Health Services Administration, 2003) and the National Survey on Drug Use and Health (Substance Abuse and Mental Health Services Administration, 2005; Substance Abuse and Mental Health Services Administration, 2007; Substance Abuse and Mental Health Services Administration, 2009; Substance Abuse and Mental Health Services Administration, 2011; Substance Abuse and Mental Health Services Administration, 2013; Substance Abuse and Mental Health Services Administration, 2015; Substance Abuse and Mental Health Services Administration, 2017; Substance Abuse and Mental Health Services Administration, 2019). **(B)** Unspecified polysubstance use in treatment-seeking drug users in Finland from 1997 to 2008. *Top*: Primary (left) and secondary (right) drugs used by treatment-seeking drug users, shown as percent of total users. *Bottom*: Percent of users reporting exclusive misuse of one drug (white bars) or misuse of a given drug along with polysubstance use of another (colored bars). Data from Onyeka et al. (2012).

Drug addiction is a heterogeneous disorder characterized by cyclic periods of drug use, withdrawal and abstinence, and drug-craving and recurrence of use (Koob and Volkow, 2016). Addiction is highly prevalent in our society, with an estimated 35 million people world-wide and 19.3 million people in the United States (US) currently meeting diagnostic criteria for a substance use disorder (SUD) (Figure 1A; Substance Abuse and Mental Health Services Administration, 2019; United Nations Office on Drugs and Crime, 2019). Additionally, epidemiological surveys suggest that, in a person’s lifetime, there is a ∼10% prevalence of a SUD (Grant et al., 2016; Substance Abuse and Mental Health Services Administration, 2019). Drug addiction is also one of the largest public health problems in the US, with an annual financial burden of $740 billion in costs related to treatment, lost work productivity, healthcare, and crime (National Institute on Drug Abuse, 2020). These numbers are likely to increase as illicit drug use is rising, with a quarter of a billion people worldwide reporting use in the past year (United Nations Office on Drugs and Crime, 2019). Within the US, over 17 million people aged 12 and above are estimated to initiate drug use annually. Rates of opioid use, in particular, are continuing to climb, with 53 million past-year opioid users worldwide and ∼11 million people in the US reporting opioid misuse within the past year (United Nations Office on Drugs and Crime, 2019). This is especially alarming, as the number of deaths in the US involving opioids has increased 6-fold from 1999 to 2017, with ∼130 Americans dying from use per day (Centers for Disease Control, 2018).

Although the majority of research on SUDs has focused on individual substances in isolation, with a multiple drug use history often considered an exclusion criterion for clinical studies, it is important to recognize that many drug users engage in polysubstance use. For instance, 30–80% of heroin users have been reported to also use cocaine (Leri et al., 2003a), and deaths involving both cocaine and opioids in the US more than doubled between 2010 and 2015 (Centers for Disease Control, 2018). A person is considered a polysubstance user if they use more than one substance, including use of multiple drugs on separate occasions (sequential use) or at the same time (concurrent/simultaneous). Limiting studies to individual drugs risks overlooking interactions between substances, decreases translatability of preclinical research, and can impede the efficacy of identified treatments for SUDs. Indeed, polysubstance use has consistently been associated with worse treatment outcomes, including poorer treatment retention, higher rates of relapse, and a three-fold higher mortality rate compared to mono-substance use (Williamson et al., 2006; Staiger et al., 2013; de la Fuente et al., 2014). This review seeks to combine the current knowledge of the mechanisms and consequences of individual drug use with the most up-to-date research on polysubstance use, making sure to note, when possible, if polysubstance use is concurrent/simultaneous, sequential, or a combination of these patterns. We will first provide an overview of public health trends regarding single and polysubstance use, as well as the impact of polysubstance history on metrics of substance use severity. This will be followed by a discussion of findings from preclinical studies, and their translatability to real-world substance use, outlining considerations to be made when designing polysubstance studies. We then detail the pharmacology of individual substances and some of their effects on the cortical-basal ganglia-thalamic (C-BG-T) circuitry, which sets the groundwork for understanding how polysubstance use may change the neuropathology of addiction. Care will be given to discussing the differences between brain alterations in single versus polysubstance use, highlighting the most common combinations of polysubstance use. For clarity and in order to avoid duplication in our discussions, sections are organized by primary used substance, with consideration for the consequences that result when additional drugs are combined with a primary drug. Finally, we offer suggestions and highlight potential methods to move forward with the important task of examining polysubstance disorders.

## Public Health Trends in Substance Use

Drug addiction is both pervasive and deadly, with ∼585,000 drug use-related deaths occurring each year worldwide (United Nations Office on Drugs and Crime, 2019). Nonetheless, although drug addiction and its impacts are often centered around individual drugs, drug misuse is largely found to involve multiple substances (Gjersing et al., 2013; Roy et al., 2013; Substance Abuse and Mental Health Services Administration, 2016). Indeed, drug-dependent individuals report an average use of 3.5 substances (Onyeka et al., 2012), including both simultaneous and sequential polydrug use (Figure 1B). In addition, the likelihood of developing comorbid substance dependencies is high in clinical populations (Leri et al., 2004; Lorvick et al., 2018). Although combinations of co-used substances vary, primary drug dependencies are typically found for alcohol, opioids, amphetamine, and methamphetamine, while cannabis and cocaine are more often reported as secondary-or tertiary-used substances (Substance Abuse and Mental Health Services Administration, 2016). The high prevalence of polysubstance use is particularly concerning given the impact it can have on both SUD severity and treatment outcomes. For example, a polysubstance history is associated with greater unmet physical and mental health care needs, increased risk behavior, violence, and increased overdose and mortality risk compared to single substance use (Pennings et al., 2002; Gilmore et al., 2018; Lorvick et al., 2018). In this section, we will discuss the public health consequences surrounding single substance use, as well as polysubstance use in relation to secondary substance combinations. This overview will aim to address overarching patterns of polydrug use, including the substance combinations and patterns of use that most commonly occur. However, given that data is limited for specific drug use patterns, types of classification, and differences in definition of polydrug combinations across studies, it is unlikely to capture all combinations, histories, and patterns of use.

### Psychostimulants

Psychostimulants are the second-most widely used class of drugs, with 18 million current cocaine users and 29 million current prescription stimulant users worldwide (United Nations Office on Drugs and Crime, 2019). Worldwide prevalence of psychostimulant use has remained relatively stable from 1990 to 2017, with 7.38 million reported to meet criteria for an amphetamine use disorder and 5.02 million reported to meet criteria for a cocaine use disorder (Degenhardt et al., 2018). However, the number of drug-related overdose deaths involving psychostimulants has continued to climb, especially in the US, with a 2.6-fold increase in the cocaine overdose death rate and 3.6-fold increase in the methamphetamine overdose death rate from 2000 to 2017 (Degenhardt et al., 2018).

Notably, cocaine and amphetamine users are predominantly polysubstance users, with one study reporting 74 and 80% incidence of polysubstance history, respectively (Kedia et al., 2007). Specifically, cocaine use and developing a cocaine use disorder is associated with concurrent heroin, cannabis, tobacco, and alcohol use (Kedia et al., 2007; Roy et al., 2013; John and Wu, 2017). Similarly, amphetamine users exhibit several types of polysubstance use, with high probabilities of alcohol, tobacco, and cannabis use. In addition, other classes of amphetamine polysubstance users exhibit higher probabilities of heroin and other opioid use. Across groups, lower probabilities of cocaine use with amphetamine compared to other drug classes used with amphetamine are reported as well (Darke and Hall, 1995; Kelly et al., 2017). Polysubstance use is common among stimulant users with both concurrent and sequential drug consumption patterns. For instance, simultaneous use of psychostimulants and opioids is seen with both cocaine (“speedball”) and methamphetamine (“bombita”). Sequential use of psychostimulants and opioids is also common, including the use of cocaine or amphetamine to avoid opioid-related somatic withdrawal symptoms (Hunt et al., 1984; Ellis et al., 2018) and the use of opioids to reduce overexcitation following cocaine use (Kreek, 1997). Additionally, there is an increased likelihood of same-day methamphetamine use with alcohol consumption (Bujarski et al., 2014).

Though these studies did not specify the patterns of polydrug use, a meta-analysis of reports on concurrent versus simultaneous cocaine use found a 24–98% range of simultaneous cocaine and alcohol use and 12–76% incidence of simultaneous cannabis use (Liu et al., 2018). Rates of concurrent use were 37–96% for cocaine and alcohol use, 43–94% for cocaine and cannabis use (Liu et al., 2018), 70–80% for cocaine and nicotine use (Budney et al., 1993; Weinberger and Sofuoglu, 2009), and 85–95% for amphetamine and nicotine use (Brecht et al., 2007; Grant et al., 2007). The high variability in reported frequencies highlights the complexity in identifying drug use patterns, which can vary across demographics, study periods, study structure, and definitions of concurrent and simultaneous use.

Polydrug use involving psychostimulants poses significant public health risks. For example, one study showed that amphetamine users were 21 times more likely to have a concurrent cannabis use disorder and 7 times more likely to have past-year concurrent cocaine use, compared to those with no prior history of amphetamine use (Massaro et al., 2017). In addition, nearly one-third of overdose deaths involved both psychostimulants and opioids, such as heroin and fentanyl (Kariisa et al., 2019). The hazards of psychostimulant co-use also extend to other substances, with combined cocaine and cannabis use resulting in higher standardized death rates in emergency department (ED) visits, suggesting elevated mortality risks with this combination (Gilmore et al., 2018). Additionally, combining cocaine and alcohol use increases the risk for cardiotoxicity compared to either drug alone (Pennings et al., 2002).

### Nicotine

Although the use of tobacco (i.e. the dried leaves of the tobacco plant containing nicotine) has declined since the early 2000s, nicotine is still one of the most commonly used drugs, with 58.8 million people aged 12 or above reporting past-month nicotine use in the US (Substance Abuse and Mental Health Services Administration, 2019). It is also commonly used with many other substances, as 17% of nicotine users also used cannabis, 4.7% also used opioids, 2.6% also used cocaine, and 1.4% also used psychostimulants in the past month. In contrast, nonsmokers had much lower percentages of past-month substance use (3.7, 1.2, 0.2, and 0.3% for the aforementioned substances, respectively), (Moeller et al., 2018). This difference is notable, given that people with a nicotine use disorder are 3–4 times more likely to have a second SUD (Chou et al., 2016). In addition, it was found that past-year tobacco use was significantly associated with opioid use disorders, as well as comorbidities for cannabis and alcohol use and use disorders, and cocaine use in samples of primary care patients (John et al., 2019). Tobacco use severity (i.e. frequency of use and number of cigarettes smoked) has also been significantly correlated with onset of heroin and cocaine use (Frosch et al., 2000). Historically, nicotine has primaryily been used by smoking tobacco cigarettes. However, new advances in technology have led to the development of electronic (e-) cigarettes, designed to deliver nicotine in a toxin-free manner. The marketing of e-cigarettes as a safer alternative to traditional tobacco cigarettes is concerning, as it has led to an increase in the probability of nicotine use and a resurgence in the potential for nicotine addiction. For example, e-cigarette use among middle and high school students has increased from 2012 to 2016 (Gentzke et al., 2019), and a spike in use was observed among young adults (18–24 years old) around 2013 to 2014, when e-cigarette products were introduced (Gentzke et al., 2019). Despite delivery of lower doses of nicotine, the safety of commercial e-cigarettes has been debated, since compensatory “puffing” behaviors or high voltage settings leads to the production of carcinogenic agents (Jensen et al., 2015). The potential danger of use is further compounded by the variable amounts of nicotine provided across e-cigarette manufacturers (Goniewicz et al., 2013). The unique influence of vaping on the development of nicotine dependence and how this differentially contributes to polysubstance use disorders remains largely unknown and should be studied in the coming years.

### Opioids

The prevalence of opioid misuse (i.e. use outside of prescribed use) has risen dramatically in recent years, with ∼53 million adults (1.1% of global population) reporting past-year non-medical use of an opioid (United Nations Office on Drugs and Crime, 2019). In the US alone, 11 million people reported past-year opioid misuse in 2016 (Substance Abuse and Mental Health Services Administration, 2017); however, this estimate is conservative as it does not include homeless or incarcerated individuals with disproportionately higher levels of opioid use. In addition, the rate of first-time heroin users rose in parallel with non-medical use of prescription opioids from 2002 to 2011 (United Nations Office on Drugs and Crime, 2019), a reflection that suggests individuals with past-year prescription opioid misuse are 19 times more likely to initiate heroin use than those without such a history (Muhuri et al., 2013; Cicero et al., 2018). Studies have investigated polydrug use among heroin and prescription opioid misusers and found higher frequencies of opioid use in people that also use cocaine (>33%) or methamphetamine (>20%) (Wang et al., 2017; Hedegaard et al., 2018), but reduced prevelance for primary opioid use in those that have secondary alcohol or cannabis use (Wang et al., 2017; but see Cicero et al., 2020). In addition, first-time methamphetamine use is more prevalent following past-month opioid use (Cicero et al., 2020). Of those entering treatment for heroin use, it has been found that 91% of people reported a lifetime history of cocaine use (Williamson et al., 2006). Additionally, a study in the United Kingdom found that 54% of opioid users in treatment between 2017 and 2018 also had a comorbid crack cocaine use disorder (Public Health England, 2018). With respect to patterns of multi-drug use, simultaneous use of heroin with alcohol and/or cannabis is more common than with psychostimulants (Kelly et al., 2017; Bobashev et al., 2018), and a sequential pattern of drug use is preferred for opioids and psychostimulants.

In the US, opioid use is a national public health emergency responsible for more than 1.6 million years of life lost from 2001 to 2016 (Gomes et al., 2018). Moreover, opioid overdose deaths are currently the leading cause of accidental death among US adults, with 68% of all drug overdose deaths involving an opioid (United Nations Office on Drugs and Crime, 2019). Given that nearly 80% of fatal opioid overdoses also involved another substance, it appears that there is a greater risk of death when opioids are used in combination with other opioids and/or other drugs (Jones et al., 2018). Specifically, of these deaths, 78% involved another opioid, 21.6% involved cocaine, 11.1% involved alcohol, and 5.4% involved a psychostimulant other than cocaine (Jones et al., 2018). Furthermore, opioid-related ED visits also involved tobacco (51.1%), cocaine (36.9%), other stimulants (22.6%), cannabis (25.1%), or alcohol (16.9%). Substantial polysubstance use of three or more of these substances has also been reported for opioid-related ED visits (Liu and Vivolo-Kantor, 2020), and the likelihood of these visits has been associated with the degree of severity of other SUDs (Zale et al., 2015; John et al., 2019). Taken together, these reports suggest that combining opioid use with use of other substances can exacerbate the deleterious consequences of opioid use. In addition to overdose risk, opioid users experience very high rates of relapse, with 59% of individuals relapsing in the first week and 80% relapsing in the first month of abstinence (Smyth et al., 2010). Past use of other substances, including the degree of cocaine use, increases relapse susceptibility (Williamson et al., 2006). Methamphetamine use among those seeking treatment for opioid use has also been on the rise (United Nations Office on Drugs and Crime, 2019), and recent reports indicate that methamphetamine use is associated with a discontinuation of buprenorphine treatment in people with an opioid use disorder (Tsui et al., 2020). Thus, a better understanding of the impact of polysubstance use in the context of opioids is crucial for more successful emergency responses and long-term treatment outcomes.

### Cannabinoids

It is estimated that 188 million individuals 12 years or older use cannabis worldwide (United Nations Office on Drugs and Crime, 2019), including 43.5 million individuals in the US (Substance Abuse and Mental Health Services Administration, 2019). Beginning in 2012 with Washington and Colorado, 11 states and the District of Columbia have legalized recreational cannabis, making it legally accessible to ∼328 million people. The number of cannabis users in the US has risen with its gradual decriminalization and legalization, from 4.1% in 2002, to 9.9% in 2007, to 15.9% in 2018 (Hasin et al., 2015; Substance Abuse and Mental Health Services Administration, 2019). Frequency of cannabis use is also high, with reports of 40% of individuals being daily or near-daily users (Substance Abuse and Mental Health Services Administration, 2019).

Cannabis users are reported to have high rates of past month tobacco, alcohol, and/or amphetamine use (Connor et al., 2013). One of the most common combinations is simultaneous use of alcohol and cannabis (McCabe et al., 2006), along with simultaneous alcohol, cocaine, and cannabis use (Liu et al., 2018). The impact of concurrent cannabis is notable, as this pattern of use is associated with more alcoholic drinks per day, suggesting facilitation of alcohol use with coexisting cannabis consumption (Aharonovich et al., 2005; Subbaraman et al., 2017). Polydrug use is particularly prevalent in younger populations. Among adolescent cannabis users, 27.5% reported additional drug use within the same year of starting cannabis use, and nearly 67% use two or more other drugs (Subbaraman and Kerr, 2015). Cannabis is frequently used during treatment for other SUDs (Connor et al., 2013; Subbaraman et al., 2017), and this has been associated with reduced treatment efficacy. For example, cannabis use has been found to result in shorter periods of alcohol abstinence (Subbaraman et al., 2017), as well as greater incidence of relapse to cocaine (Aharonovich et al., 2005; Mojarrad et al., 2014). In addition, polydrug use among cannabis users has been correlated with reduced socioeconomic mobility, financial instability, and relationship difficulties (Aharonovich et al., 2005; Cerdá et al., 2016; Subbaraman et al., 2017), a heightened degree of mood disorder symptom severity, decision-making deficits, social difficulties, and self-harm (Subbaraman and Kerr, 2015; Lopez-Quintero et al., 2018). Although these data suggest that the consequences of drug use are enhanced by concurrent cannabis use, it should be noted that clinical outcomes can vary for studies examining polydrug use among cannabis users. For example, some studies suggest a nuanced impact of polysubstance use that is dose-dependent, with no synergistic effects of cannabis and alcohol at low doses of either drug (Ballard and De Wit, 2011), and a lack of association of cannabis use in heroin relapse (Aharonovich et al., 2005).

### Alcohol

Alcohol is one of the most commonly used drugs, with up to 290 million people diagnosed with an alcohol use disorder worldwide (United Nations Office on Drugs and Crime, 2019), including 15 million people in the US (Substance Abuse and Mental Health Services Administration, 2019). Alcohol is frequently used with other substances, with reports indicating that 5.6% of US adults have used both alcohol and another illicit drug within the past year, and 1.1% have met diagnostic criteria for both an alcohol use disorder and another SUD (Falk et al., 2006). The most commonly reported substance co-used with alcohol is cannabis (10%), with less common comorbidities found with opioids (2.4%), cocaine (2.5%), and amphetamine (1.2%) (Falk et al., 2006). Although simultaneous use of alcohol and cannabis or alcohol and prescription opioids is most common (McCabe et al., 2006), simultaneous use is also seen with cocaine (Liu et al., 2018).

Polydrug use increases the risk of developing an alcohol use disorder (Grant et al., 2015, 2016), particularly in young adults, men, and American Indians/Alaskan Natives (Falk et al., 2006). Polydrug use that includes alcohol is associated with additional comorbidities, including higher prevalence of mood disorders, anxiety disorders, more intense drinking, and more intense drug consumption and drug-craving (Preston et al., 2016; Saha et al., 2018). The negative consequences of alcohol polydrug use are also highlighted by data indicating that 21% of ED visits for patients 12–24 years old involved both alcohol and drugs. These visits were also more likely to require treatment for injuries, and had higher rates of inpatient admittance (Naeger, 2017). In addition, 17% of substance treatment admissions were related to both alcohol and drug use, representing 45% of primary alcohol admissions and 33% of drug misuse admissions (White et al., 2011; Substance Abuse and Mental Health Services Administration, 2016; Naeger, 2017). The rate of hospitalizations involving alcohol polydrug use has been increasing, particularly in young adults, with reports suggesting a 76% rise in inpatient admittance between 1998 and 2008, compared to either drug or alcohol overdoses alone (White et al., 2011). While the polysubstance users in these surveys were primarily white and male, recent trends indicate a rise in ED visits relating to alcohol and drug combinations in females (Naeger, 2017), suggesting a change in the demographics of polysubstance combinations that include alcohol.

## Behavioral Models of Addiction in Polydrug Studies

Behavioral models of drug addiction are used to examine the neurobiological underpinnings of the development, maintenance and relapse to drug use. The most commonly used models are locomotor sensitization (a progressive and persistent increase in locomotor responses to the same dose of a drug), conditioned place preference (CPP; a test of drug reward measured as an increase in time spent in a drug-paired chamber) and drug self-administration (response-contingent intake of drug) (see Panlilio and Goldberg, 2007; Spanagel, 2017; Kuhn et al., 2019 for review). Experimental designs using these models vary across a number of pharmacological and non-pharmacological parameters including contingency of drug use, amount of access to drug, context associated with drug use, and routes of administration. Here, we describe how these models have been used with polydrug combinations, and how this work has informed our understanding of polydrug use and addiction.

Initial polysubstance studies largely used noncontingent models of drug administration, particularly CPP and cross-sensitization models, whereby the impact of priming doses of one drug on side preference or motor activity, respectively, of another drug are determined (Shippenberg et al., 1998; Lu et al., 2002; Cole et al., 2003; Leri et al., 2003b; Liang et al., 2006). More recently, studies have been examining how drug self-administration history impacts subsequent drug choice preference and/or drug-craving via responding to drug-associated cues following extinction and/or withdrawal (Leri and Stewart, 2001; De Luca et al., 2019; Rubio et al., 2019; Crummy et al., 2020). Preclinical polysubstance models involving simultaneous administration of multiple drugs, such as alcohol and nicotine or cocaine and heroin combinations, have also been used frequently (Mello and Newman, 2011; Mello et al., 2013; Sentir et al., 2020). In clinical models, both concurrent and sequential polysubstance use is assessed in subjects via scoring of affective measures to drug-taking, drug-craving following visual cues, and autonomic response measures such as blood pressure and heart rate (Foltin et al., 1993; Greenwald et al., 2010; Giasson-Gariépy et al., 2017).

More recently, studies are comparing single versus polysubstance self-administration to determine the effect of drug history on drug-induced molecular and circuit alterations (Briggs et al., 2018; Stennett et al., 2020; Zhu et al., 2020). Additionally, paradigms based on behavioral economic principles can determine the preferred level of drug intake (i.e. no-cost intake; Q_0_), as well as the amount of effort an animal is willing to exert to defend Q0 before consumption and responding begins to decline (i.e. price; P_max_) (Oleson and Roberts, 2009). These paradigms are especially powerful in that they can use Q_0_ and P_max_ to generate normalized measures of value (i.e. essential value; α) and price (_n_P_max_), which have been used to compare price sensitivity, effort, and value across different drug and non-drug rewards in polysubstance models in several species (e.g. in rats, rhesus monkeys, and human participants) (Petry and Bickel, 1998; Ward et al., 2006; Wade-Galuska et al., 2007, 2011; Crummy et al., 2020). In particular, these studies permit examination of the relative reinforcing properties of different doses and classes of drugs (Wade-Galuska et al., 2007; Cooper et al., 2010; Huskinson et al., 2015), as well as alterations in cost valuation for a drug following pre-exposure to another drug (Cooper et al., 2010; Hofford et al., 2016; Morris et al., 2018). Direct quantification of the assigned value of these drugs across different polysubstance histories and drug doses is very useful for assessing the impact of polysubstance history on relative reinforcer value. Furthermore, these measures can be used to compare how polysubstance users value drug rewards across different experimental parameters (e.g. differences in priming dose of one drug, environmental context, pattern of drug use). Finally, clinical studies are using questionnaires or controlled laboratory environments to investigate the behavioral effects of a polysubstance history. Specifically, these studies use monetary choice procedures that compare assigned value of drugs at different doses (Greenwald et al., 2010), how assigned value changes for one drug with a change in price of another (Petry and Bickel, 1998; Petry, 2001; Sumnall et al., 2004; Chalmers et al., 2010), or how relative value of one drug changes with perceived change in subjective quality of another available drug (Cole et al., 2008). Additionally, progressive ratio tests for a single drug or drug combinations to study motivation (Greenwald et al., 2010), and delay-discounting rates for money and drug rewards to study decision-making, (Strickland et al., 2019) have also been performed. These studies permit comparison of perceived value across multiple drugs in participants with histories of single or polysubstance use (for further review, see Heinz et al., 2012).

### Effects of Polydrug Use on Addictive Behaviors

**FIGURE 2:**
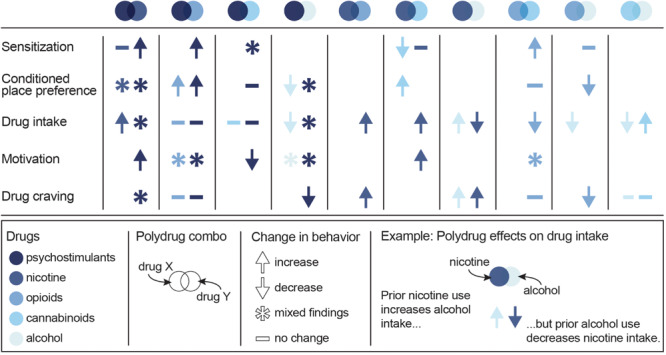
Summary of the effects of specific polydrug combinations on assays of addiction-like behaviors. Studies are organized into X/Y polydrug combos (columns) and behavioral assays (rows), with subcolumns for the effect of drug Y on drug X (left subcolumn) and the effect of drug X on drug Y (right subcolumn). Symbols represent the net effect of the X/Y polydrug combo on a given behavior, with color depicting the specific drugs tested. Sensitization: locomotor sensitization; Conditioned place preference: acquisition, expression; Drug intake: self-administration; Motivation: progressive ratio, behavioral economics; Drug craving: reinstatement, cue reactivity. Data from Mello and Mendelson (1978), Mello et al. (1980), Mello et al. (2014), Huston-Lyons et al. (1993), Foltin et al. (1993), Aspen and Winger (1997), Ranaldi and Wise (2000), Valverde et al. (2001); De Vries et al. (2001), Parker et al. (2004); Solinas et al. (2005), Liang et al. (2006); Biala and Budzynska (2006), Ward et al. (2006); Panlilio et al. (2007), Panlilio et al. (2013), Winger et al. (2007), Lê et al. (2010), Lê et al. (2014), Levine et al. (2011), Cortright et al. (2011), Pomfrey et al. (2015), Maguire and France (2016); Mahmud et al. (2017), Fredriksson et al. (2017); Giasson-Gariépy et al. (2017), Griffin et al. (2017); Schwartz et al. (2018), Winkler et al. (2018); Manwell et al. (2019), Ponzoni et al. (2019), Crummy et al. (2020), and Stennett et al. (2020).

Given the unique neurobiological alterations that can occur with exposure to multiple drugs, along with the high prevalence of polysubstance use disorders, there is a strong need to develop polydrug paradigms that have high translational value. These paradigms are critical for fully understanding the behavioral changes and addiction-related phenotypes that develop following polydrug use. However, given the vast number of potential substance combinations and the variability in methodologies that exist across studies, there are currently mixed results and interpretations regarding the impact of polydrug history on addiction-related behaviors. Nonetheless, some general trends in drug consumption, drug preference and drug-seeking have been demonstrated in commonly investigated substance combinations (Figure 2).

#### Psychostimulants

Some of the more commonly studied polydrug combinations include administration of cocaine with other drugs (Francesco et al., 2003; Leri et al., 2003b; Substance Abuse and Mental Health Services Administration, 2016). However, in contrast to human reports (Heil et al., 2001; Williamson et al., 2006; Staiger et al., 2013; Preston et al., 2016; Lorvick et al., 2018; Kariisa et al., 2019), an increase in addiction-like severity has not been observed in preclinical studies, suggesting a need for models with greater translational relevance that can capture the enhanced severity seen in human polysubstance users. Specifically, animal studies of sequential cocaine and alcohol or cocaine and heroin use have not found differences in drug intake or reinstatement of drug-seeking as a function of single versus polydrug use (Pattison et al., 2014; Fredriksson et al., 2017; Crummy et al., 2020; Stennett et al., 2020). These effects were observed despite variance in the use of contingent and non-contingent drug administration, drug doses, and species, including rats (Crummy et al., 2020; Stennett et al., 2020) and rhesus monkeys (Aspen and Winger, 1997). Consistent with this work, intermittent alcohol exposure has not been shown to affect cocaine self-administration (Aspen and Winger, 1997; Fredriksson et al., 2017) or the reinforcing properties of cocaine measured via demand curves in rhesus monkeys (Winger et al., 2007). In addition, intermittent alcohol exposure has not been shown to affect progressive ratio tests of motivation for cocaine (Mateos-García et al., 2015), or the long-term reconsolidation of preference for cocaine in drug-paired contexts (Zhu et al., 2020) in rats. In contrast, adolescent alcohol exposure has been shown to have long-lasting effects on cocaine self-administration and reward, suggesting that this population is particularly susceptible to the effects of polysubstance use. For example, adolescent alcohol exposure increases motivation for cocaine (Mateos-García et al., 2015), enhances the development of a cocaine CPP in both mice (Molet et al., 2013) and rats (Hutchison and Riley, 2012; Mateos-García et al., 2015), and weakens cocaine-induced taste aversion (Busse et al., 2005). In addition, simultaneous heroin and psychostimulant administration increases the motivation to self-administer cocaine (Ward et al., 2006) and methamphetamine (Ranaldi and Wise, 2000), and both simultaneous and sequential administration of morphine and methamphetamine have been shown to be rewarding, as measured by the development of a CPP (Briggs et al., 2018). Pretreatment with an opioid also enhances methamphetamine-induced psychomotor sensitization (Liang et al., 2006). These findings suggest that opioids can enhance the rewarding and motivational properties of psychostimulants, particularly when administered simultaneously.

Polydrug studies with cocaine and nicotine have largely reported additive and/or synergistic effects of the two drugs. In particular, co-administration of cocaine and nicotine increases drug intake in rhesus monkeys (Mello et al., 2014) and rats (Bechtholt and Mark, 2002), enhances locomotor sensitization and the development of a CPP in mice (Levine et al., 2011), and induces a cross-sensitized drug-craving (Reid et al., 1998; Weinberger and Sofuoglu, 2009; Cortright et al., 2012). Additionally, chronic nicotine pretreatment facilitates the acquisition of cocaine self-administration (Horger et al., 1992; Bechtholt and Mark, 2002; Linker et al., 2020), increases motivation under a progressive ratio schedule, impairs extinction learning, and enhances drug-primed reinstatement for amphetamine following amphetamine self-administration (Cortright et al., 2012). Conversely, prior nicotine treatment history reduces demand elasticity for cocaine (Schwartz et al., 2018). Notably, the effects of nicotine and psychostimulant polydrug use are largely dose-dependent as pretreatment with a smaller dose of nicotine [0.3 mg/kg, subcutaneous (*sc*)] increases motivation to take cocaine under a progressive ratio schedule in rats, whereas a larger dose of nicotine (0.6 mg/kg, *sc*) has the opposite effect (Bechtholt and Mark, 2002). Additionally, simultaneous administration of methamphetamine [2.0 mg/kg, intraperitoneal (*ip*)] and nicotine (1.0 mg/kg *ip*) induces a conditioned place aversion in mice, while sequential administration of the same dose induces a CPP (Briggs et al., 2018). Together, these studies demonstrate that the effects of nicotine on psychostimulant motivation, intake, and reward are heavily impacted by the parameters surrounding nicotine delivery (e.g. dose, route, pattern, etc.), which should be carefully considered when comparing study results and developing preclinical polydrug paradigms. Notably, adolescent exposure to nicotine has no effect on the subsequent development of a cocaine CPP, cocaine-induced taste aversion, cocaine self-administration, extinction, or reinstatement of cocaine-seeking in adulthood (Pomfrey et al., 2015), although it has been shown to enhance cocaine self-administration in adolescent rats (Linker et al., 2020). These data suggest that, unlike alcohol, early exposure to nicotine does not lead to increases in addiction-like behavior to cocaine in animals.

In humans, simultaneous cocaine and cannabis use produces feelings of “stimulated” and “high” that last longer than either drug alone (Foltin et al., 1993), and cue-induced drug-craving in individuals who co-use cocaine and cannabis lasts longer than for those who only use cocaine (Giasson-Gariépy et al., 2017). In contrast, THC reduces the motivation to self-administer cocaine in rodents (Panlilio et al., 2007). Although this suggests a differential regulation of cocaine’s effects in humans and rodents, further work is necessary to ensure that animal models of increased addiction severity cannot, in fact, be developed. Notably, however, neither cocaine and cannabis nor cocaine and alcohol co-administration in humans produces subjective effects that are different from cocaine, cannabis, or alcohol alone (Foltin et al., 1993). Similarly, THC pretreatment in rodents has no effect on psychostimulant reward or self-administration, nor does it potentiate the development of a CPP to amphetamine (Panlilio et al., 2007; Cortright et al., 2011; Keeley et al., 2018), indicating a unique effect of cocaine and cannabis on drug-craving. Interestingly, CBD has no effect on cocaine self-administration, motivation, or cue-induced reinstatement of cocaine-seeking (Mahmud et al., 2017), suggesting the effects of cannabis on cocaine craving are likely due to THC, rather than CBD. However, CBD treatment has been found to reduce motivation to self-administer methamphetamine on a progressive ratio schedule and to reduce methamphetamine-primed reinstatement of drug-seeking (Hay et al., 2018). The additive effects of cannabinoid and psychostimulant polydrug use appear to be dependent on both the amount of drug consumed and the age range during use. For example, acute THC weakens psychomotor sensitization, but repeated THC administration promotes tolerance to the acute effects, increasing amphetamine-induced stereotypy and locomotor activity (Gorriti et al., 1999; Cortright et al., 2011). Additionally, adolescent THC exposure accelerates acquisition of cocaine self-administration and increases intake of low doses of cocaine (Friedman et al., 2019), indicating long-lasting changes in reward circuitry following adolescent THC use, similar to alcohol.

#### Nicotine

Limited work has focused on the effects of polydrug use on nicotine-induced addiction behaviors. However, it has been found that THC pretreatment can enhance nicotine consumption and price inelasticity (measured by α) in behavioral economic tests in rats (Panlilio et al., 2013), and heroin intake has been found to increase cigarette consumption in people (Mello et al., 1980). In addition, pre-exposure to alcohol or simultaneous access to both alcohol and nicotine decreases nicotine self-administration in rodent studies (Lê et al., 2010, 2014). Although access to alcohol has no effect on responding for nicotine under extinction conditions, a priming dose of alcohol does reinstate nicotine-seeking (Lê et al., 2010). Finally, systemic co-administration of methamphetamine and nicotine produces a conditioned place aversion in rats (Briggs et al., 2018), whereas pretreatment with either amphetamine or morphine increases the rewarding properties of nicotine as shown with lowered intracranial self-stimulation thresholds (Huston-Lyons et al., 1993). These studies further emphasize the need to consider use patterns and dose in interpretation of polydrug use effects.

#### Opioids

Similar to psychostimulant polydrug studies, sequential use of heroin and cocaine has not been found to alter heroin self-administration or reinstatement of heroin-seeking (Crummy et al., 2020). Although alcohol pretreatment can prevent the long-term reconsolidation of preference for morphine in drug-paired contexts (Zhu et al., 2020), adolescent alcohol exposure enhances the development of a morphine CPP (Molet et al., 2013). This finding indicates that the long-term effects of adolescent alcohol exposure are generalizable to multiple drug classes. Interestingly, co-administration of morphine and THC prevents the development of the analgesic tolerance that normally accompanies long-term exposure to either drug alone (Cichewicz and McCarthy, 2003; Cox et al., 2007; Smith et al., 2007). In addition, the analgesic effects of THC and oxycodone co-administration are additive to oxycodone alone (Nguyen et al., 2019). Moreover, administration of either THC or both THC and CBD attenuates naloxone-precipitated withdrawal without impacting the development of a morphine CPP (Lichtman et al., 2001; Valverde et al., 2001). These data suggest a potential role for cannabinoids in regulating a physical dependence to opioids without altering their reinforcing properties. In support of this, repeated THC administration has no effect on breakpoint during a PR test of heroin self-administration (Solinas et al., 2004, but see Nguyen et al., 2019) or relapse to heroin-seeking, although it produces a small reduction in both heroin (Maguire and France, 2016) and oxycodone (Nguyen et al., 2019) intake in fixed-ratio self-administration sessions. The effects of opioid and cannabis polydrug use, however, appear to be dose-dependent as systemic administration of THC prior to heroin self-administration reduces responding for large doses of heroin, but has no effect on responding for lower doses in both monkeys and rats (Solinas et al., 2004; Maguire and France, 2016).

#### Cannabinoids

As with psychostimulants and opioids, administration of nicotine with THC augments the effects of either drug alone when measured in tests of locomotion, analgesia, and hypothermia (Valjent et al., 2002). In addition, THC and nicotine co-administration exacerbates the somatic symptoms of THC withdrawal (Valjent et al., 2002). However, after repeated nicotine treatment and 2 weeks of drug abstinence, nicotine re-administration attenuates THC-induced decreases in locomotor activity, increases in anxiety measures (when assessed in the elevated-plus maze), and changes in social interaction (Manwell et al., 2019). These findings suggest that nicotine enhances the negative symptoms of THC when administered concurrently or in close temporal proximity. Although nicotine pretreatment enhances the rewarding effects of subthreshold doses of THC (Ponzoni et al., 2019), cocaine pretreatment heightens THC-induced anxiogenic behaviors (Panlilio et al., 2007). Cannabis and alcohol polydrug use is relatively common in humans, and individuals report reduced alcohol consumption when cannabis is available (Mello and Mendelson, 1978), suggesting a role for cannabinoids in alcohol intake. However, drug-induced cognitive and physical impairments in humans, as assessed in a driving simulation, were found to be more severe after use of THC and alcohol compared to either drug alone (Downey et al., 2013). Conducting polydrug studies of combinations of THC or CBD with other drug classes is therefore necessary to understand the differential effects resulting from these drugs.

Unfortunately, due to long-term restrictions on cannabis research in the US and past difficulties in modeling cannabis use with self-administration models in rodents (Panlilio et al., 2015), much less is known about the impact of cannabis relative to other drugs. The development of novel methods of cannabis self-administration in animals, including oral self-administration of Δ9-tetrahydrocannabinol (THC)-containing gelatin (Kruse et al., 2019), self-administration of vaporized THC and cannabidiol (CBD) (Freels et al., 2020), and intravenous self-administration of THC and CBD (Neuhofer et al., 2019) will help facilitate the preclinical study of cannabis use disorder, as well as enable us to better understand the consequences of polydrug use involving cannabis.

#### Alcohol

Polydrug use of alcohol and nicotine produce mixed phenotypes in relation to addiction behaviors. For example, pre-exposure to alcohol or simultaneous access to both alcohol and nicotine increases alcohol self-administration, but not when nicotine is administered prior to alcohol each day (Lê et al., 2010, 2014). Chronic nicotine treatment also enhances alcohol preference, an effect that persists through nicotine withdrawal (Blomqvist et al., 1996). However, although access to nicotine impairs extinction learning to alcohol responding, it has no effect on reinstatement of drug-seeking, as rats respond similarly on alcohol and nicotine-associated levers following a priming injection of nicotine (Lê et al., 2010). Nonetheless, another study found that priming doses of alcohol, but not nicotine, were capable of reinstating alcohol-seeking following self-administration of both nicotine and alcohol (Sentir et al., 2020). Studies have not systematically examined the effects of other drugs on alcohol use and addiction.

## Neurobiology of Addiction

**FIGURE 3:**
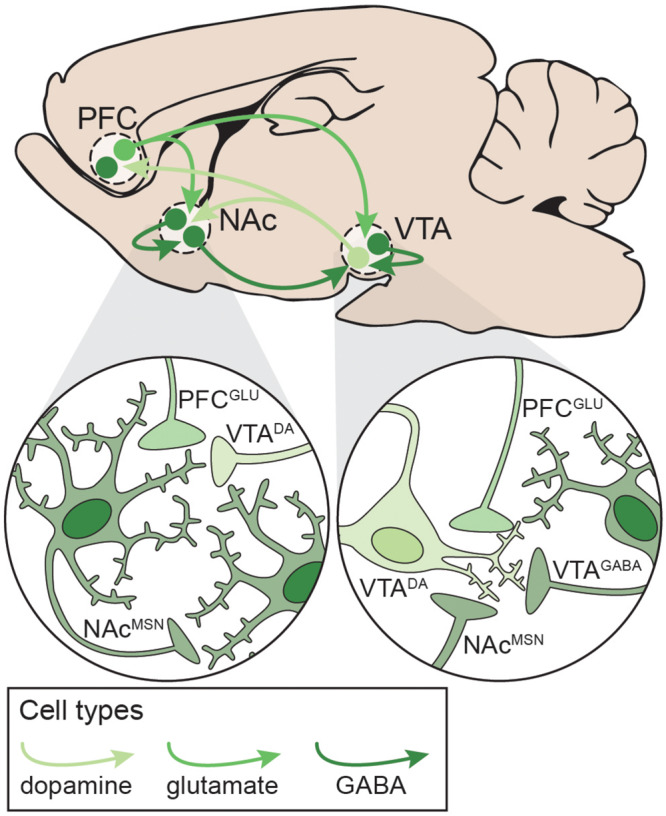
Neural circuitry targeted by potentially addictive drugs. Simplified schematic emphasizing local and distal connections between the PFC, NAc, and VTA that are targeted by potentially addictive drugs. *Left*: NAc^MSNs^ receive excitatory glutamatergic inputs from PFC^GLU^ neurons, dopaminergic inputs from VTA^DA^ neurons, and inhibitory GABAergic inputs from other NAc^MSNs^. *Right*: VTA^DA^ neurons are maintained under tonic inhibition by local VTA^GABA^ interneurons and NAc^MSNs^ and receive excitatory inputs from PFC^GLU^ neurons. DA: dopamine; GLU: glutamate; MSN: medium spiny neuron; NAc: nucleus accumbens; PFC: prefrontal cortex; VTA: ventral tegmental area.

The development and maintenance of addiction behaviors arises in part from maladaptive neuroplasticity within the neural circuits responsible for decision-making, learning, motivation, and reward processing. In particular, alterations in the cortico-basal ganglia-thalamic (C-BG-T) network are known to contribute to drug-taking and drug-seeking behaviors, as well as the persistence of SUDs (Koob and Volkow, 2016). The C-BG-T is a heavily interconnected network that integrates sensory and interoceptive cues to drive motivated behavioral output. The striatum, which serves as an interface of the C-BG-T, receives extensive glutamatergic input from cortical (e.g. prefrontal) and subcortical (e.g. amygdala, hippocampus, thalamus) regions, along with dopaminergic input from the midbrain [substantia nigra (SN)/ventral tegmental area (VTA)] (Gerfen and Surmeier, 2011; Calabresi et al., 2014). Integration of glutamatergic and dopaminergic inputs with local inhibition in the striatum contributes to the initiation or suppression of behavioral output, and imbalanced signaling between the two striatal output pathways (i.e. the direct and indirect) can drive addictive behaviors (Kravitz et al., 2010; Ferguson et al., 2011; O’Neal et al., 2019). It is beyond the scope of the current review to fully explore all of the neurobiological changes that occur in the C-BG-T with drug use. We will instead focus on one microcircuit within the C-BG-T that is central to the acute effects of drugs with addictive potential, and in some of the persistent changes that develop following long-term drug use: The prefrontal cortex (PFC) – nucleus accumbens (NAc) – VTA network (Figure 3). Following a review of the microcircuitry and connectivity of these regions, we will discuss disruptions that occur within this network following both acute and long-term exposure to different classes of drugs, with emphasis on the similarities and/or differences of effects relative to polydrug combinations.

### Prefrontal Cortex

The PFC is centrally involved in reward learning, decision-making, and outcome valuation (Garcia et al., 2018). It is a highly heterogenous structure, which adds to the complexity in understanding its role in cognition, as well as how its dysregulation contributes to drug use and addiction. In general, the medial prefrontal cortex (mPFC) – encompassing the anterior cingulate (ACC), prelimbic (PrL), and infralimbic (IL) cortices – regulates motivation and seeking of both natural and drug rewards via excitatory glutamatergic projections to the NAc and VTA (Koob and Volkow, 2016). Notably, projections from the mPFC to the NAc are topographically organized, with the PrL innervating the NAc core and the IL innervating the NAc shell (Brog et al., 1993). In contrast, the orbitofrontal cortex projects more heavily to the dorsal striatum and SN and is primarily involved in outcome and probability valuation (Padoa-Schioppa and Conen, 2017). Hypoactivity in the PFC contributes to drug craving and seeking despite negative consequences in preoccupation stages of addiction, with dysregulated connectivity to the striatum and VTA contributing to cue sensitivity and motivated drug-taking (Volkow and Boyle, 2018). The PFC is comprised of six layers, each with unique connectivity patterns and distinct cell types. Specifically, a majority of PFC neurons are large pyramidal output cells (75%), as well as several subtypes of interneurons (∼25%) (Santana and Artigas, 2017). Pyramidal cells in layers II/III send local projections within cortex, while those in layers V-VI send projections throughout the C-BG-T, including to the striatum, midbrain, amygdala, hippocampus, and thalamus (Gabbott et al., 2005; Santana and Artigas, 2017). Pyramidal cells can be further subdivided based on physiology and connectivity (Morishima and Kawaguchi, 2006; Brown and Hestrin, 2009; Reiner et al., 2010; Shepherd, 2013; Kim E. J. et al., 2015). Recent studies have begun to characterize the anatomical, electrophysiological, and molecular profiles of each of these cell types (Kalmbach et al., 2015; Kim E. J. et al., 2015; Saiki et al., 2018; Chen et al., 2019; Winnubst et al., 2019), though how they each regulate behavior remains poorly understood. Finally, the PFC contains multiple populations of interneurons that heavily regulate cortical output via projections to both pyramidal neurons and interneurons (van Versendaal and Levelt, 2016; Batista-Brito et al., 2017).

### Nucleus Accumbens

The striatum is a heterogeneous structure comprised primarily of two interspersed populations of GABAergic medium spiny neurons (MSNs) that can bidirectionally regulate behavioral output. Direct pathway MSNs (dMSNs) express dopamine D1-like (D_1_) receptors and the neuropeptides dynorphin and substance P, project directly to the midbrain, and can promote behavioral output by serving as a “go” signal. Conversely, indirect pathway MSNs (iMSNs) express dopamine D2-like (D_2_) receptors and the neuropeptide enkephalin, project indirectly to the midbrain via the pallidum (GPe and VP), and can suppress behavioral actions by serving as a “stop” signal (Kravitz et al., 2010; Gerfen and Surmeier, 2011). Drug use promotes increased phasic dopamine from D_1_ activation, prompting reward attribution to drug use during binge/intoxication phases of the addiction cycle, conditioning, and incentive salience attribution to drug-taking contexts (Volkow et al., 2011; Koob and Volkow, 2016). The striatum contains dorsal and ventral compartments, with further subdivisions based on connectivity and function. The ventral striatum – comprised of the olfactory tubercule, NAc core, and NAc shell – receives dopaminergic modulation from the VTA and glutamatergic input from the PFC, as well as thalamic, hippocampal, and amygdala nuclei (Li et al., 2018). In general, the ventral striatum regulates motivated behavior and reward learning. However, it has been hypothesized that an ascending loop between the ventral and the dorsal striatum facilitates information consolidation during learning, whereby habitual behaviors transition from the ventral striatum to the dorsal striatum, contributing to compulsive drug-seeking (Dobbs et al., 2016; Koob and Volkow, 2016; Burke et al., 2017). Importantly, while dMSNs and iMSNs have historically been differentiated by downstream targets and expression of dopamine receptors, ventral striatal dMSNs send collaterals to the VP (Cazorla et al., 2014; Kupchik et al., 2015), and D_1_ and D_2_ receptors are co-expressed to some degree in the NAc core (6–7%), and NAc shell (12–15%) (Bertran-Gonzalez et al., 2008; Gagnon et al., 2017). In addition to MSNs, the striatum contains large, tonically active cholinergic interneurons and multiple subtypes of GABAergic interneurons with distinct electrophysiological properties and peptide expression patterns (Burke et al., 2017). MSNs also receive cholinergic modulation from other projections (Dautan et al., 2014), though the relevance of these inputs to local or network dynamics and the role of cholinergic striatal neurons in the addiction cycle remains uncertain. Interestingly, each MSN receives ∼5000–15000 excitatory inputs in addition to ∼1200–1800 GABAergic inputs from other MSNs, therefore, modulation of MSN activity via cholinergic and dopaminergic inputs appears necessary for signal integration and effective synaptic plasticity (Moyer et al., 2007; Burke et al., 2017). Indeed, MSNs exhibit bi-stability, residing almost exclusively in either a down-state (−80 mV) or an up-state (−50 mV, near threshold) in the absence of external input. In addition, the maintenance of bi-stability and intrinsic excitability relies on the activity of cation channels that are under robust dopaminergic and cholinergic modulation (Plenz and Kitai, 1998; Moyer et al., 2007). Maintenance of intrinsic excitability within MSNs is critical for normal regulation of behavioral output, and dysregulation of striatal microcircuitry contributes to the development and expression of addiction behaviors (Bock et al., 2013; Stefanik et al., 2013; O’Neal et al., 2019).

### Ventral Tegmental Area

The VTA sends dopaminergic projections to cortical, striatal, and subcortical (e.g. hippocampus, amygdala, thalamus) areas to modulate C-BG-T network activity (Koob and Volkow, 2010). Dopaminergic projections from the VTA to the NAc regulate goal-directed behaviors and have been heavily implicated in the binge/intoxication phase of SUDs (Koob and Volkow, 2016). Recently identified subtypes of VTA^DA^ neurons with unique molecular profiles preferentially project to the NAc core or NAc shell, and regulate reward learning or motivation, respectively. However, co-activation of both populations appears to be necessary for robust reinforcement (Heymann et al., 2019). VTA^DA^ neurons receive dense glutamatergic input from the PFC and several midbrain structures, as well as GABAergic input from a variety of sources (reviewed in Morales and Margolis, 2017), including the NAc and local VTA^GABA^ neurons. VTA^GABA^ neurons maintain tonic DA levels via inhibition of VTA^DA^ neurons, and brief disinhibition of VTA^DA^ neurons results in phasic DA release into the NAc. VTA^GABA^ neurons receive glutamatergic input from the PFC and a number of subcortical nuclei, as well as GABAergic input from throughout the brain, including the NAc (Morales and Margolis, 2017). The VTA also contains glutamatergic neurons that project to striatal interneurons (Brown et al., 2012; Qi et al., 2016), though the inputs to and behavioral relevance of these neurons is unknown. Notably, the VTA contains subpopulations of DA neurons that can release glutamate and/or GABA (Kim J. I. et al., 2015; Berrios et al., 2016; Morales and Margolis, 2017), allowing the VTA to modulate local C-BG-T activity at multiple levels and time scales. Within the NAc, the activity of VTA^DA^ neurons is modulated by dopamine D_2_ autoreceptors on VTA^DA^ terminals and cholinergic interneurons (Morales and Margolis, 2017).

### Mechanisms of Addiction: Acute Drug Effects

**FIGURE 4:**
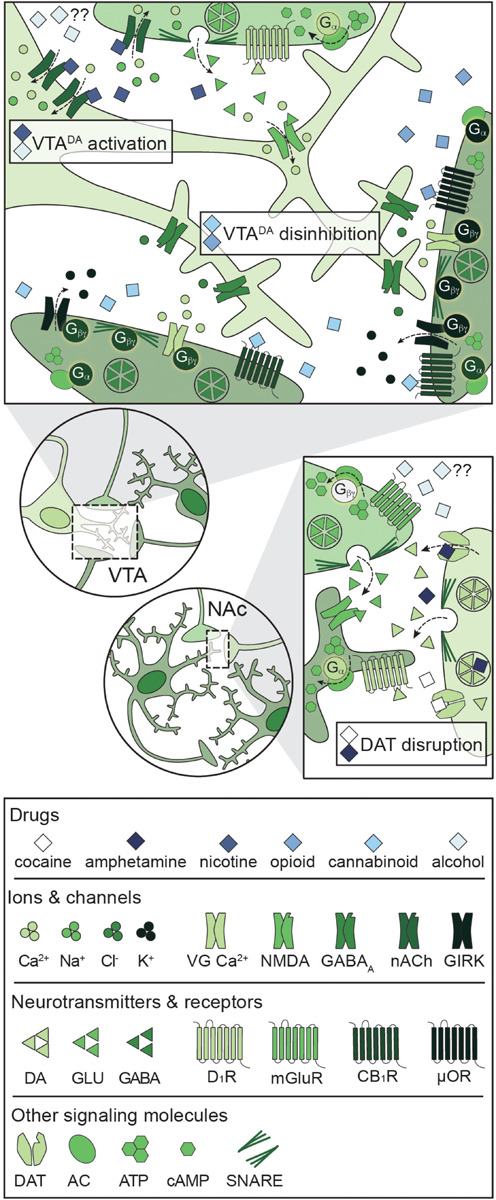
Primary mechanisms of action of potentially addictive drugs. Potentially addictive drugs increase DA release into the NAc, but different drugs act via distinct mechanisms. *Top*: Opioids and cannabinoids disinhibit VTA^DA^ neurons via presynaptic inhibition of VTA^GABA^ and NAc^MSN^ inputs through four notable mechanisms: Inhibition of VG Ca^2+^ channels, activation of GIRKs, inactivation of AC, and inhibition of GABA release. Nicotine activates VTA^DA^ neurons via direct activation of somatodendritic nAChRs and activation of presynaptic PFC^GLU^ inputs. Alcohol directly activates VTA^DA^ cell bodies, but the mechanism is not understood. *Bottom*: Psychostimulants impair DA reuptake by blocking DAT (cocaine) or reversing the activity of DAT and facilitating DA release (amphetamine), leading to increased dopaminergic tone in the NAc. Alcohol also targets PFC^GLU^ inputs to the NAc, but the net effect on PFC^GLU^ activity is unknown. AC: adenylyl cyclase; ATP: adenosine triphosphate; cAMP: cyclic adenosine monophosphate; CB_1_R: cannabinoid 1 receptor; DA: dopamine; DAT: dopamine transporter; D_1_R: dopamine D_1_ receptor; GABA: gamma- aminobutyric acid; GIRK: G protein-coupled inwardly rectifying K^+^ channel; GLU: glutamate; mGluR: metabotropic glutamate receptor; μOR: mu opioid receptor; NAc: nucleus accumbens; nACh: nicotinic acetylcholine receptor; NMDA: *N*-methyl-D-aspartate receptor; PFC: prefrontal cortex; SNARE: soluble *N*-ethylmaleimide-sensitive factor attachment protein receptor; VG Ca^2+^: voltage-gated Ca^2+^ channel; VTA: ventral tegmental area.

A unique feature of all potentially addictive drugs is the ability to reinforce the binge/intoxication phase of the addiction cycle via evoked phasic DA release into the NAc, yet the underlying mechanisms vary across drugs (Figure 4; Volkow and Boyle, 2018). Psychostimulants disrupt DA reuptake into VTA^DA^ terminals (Pontieri et al., 1995), nicotine and alcohol directly activate VTA^DA^ neurons (Pidoplichko et al., 1997; Brodie et al., 1999), and opioids and cannabinoids disinhibit VTA^DA^ neurons (Pidoplichko et al., 1997; Cheer et al., 2000). The synergistic and antagonistic interactions between different drugs within the C-BG-T lends complexity to the study of polydrug use, and little is known about the mechanisms underlying the acute effects of specific polydrug combinations. However, *in vivo* extracellular recordings in rats that alternatively self-administered cocaine and heroin in the same session found that only ∼20% of PFC and NAc neurons responded similarly to both drugs (Chang et al., 1998), indicating divergent engagement of the C-BG-T by these drugs. Thus, examining the synergistic and antagonistic mechanisms of different drugs can guide our understanding of how specific polydrug combinations may disrupt C-BG-T network dynamics and contribute to the manifestation of addiction behaviors.

#### Psychostimulants

All psychostimulants directly enhance striatal DA release via disruption of dopamine transporter (DAT) activity (Pontieri et al., 1995), though they do so via distinct mechanisms. Cocaine blocks DAT-mediated reuptake of DA, while amphetamine reverses DAT activity and induces DA release from VTA^DA^ terminals (Hyman et al., 2006; Kelly et al., 2008). Psychostimulants also acutely increase glutamate transmission in the PFC, NAc, and VTA (Reid and Berger, 1996; You et al., 2007; Shin et al., 2016), indicating broad increases in activity throughout the C-BG-T network following psychostimulant use. The combination of enhanced glutamatergic and dopaminergic input to the NAc facilitates the transition of MSNs to the up- state, and activation of DA-dependent signaling cascades (McFarland and Kalivas, 2001; Feltenstein and See, 2008). For example, psychostimulants acutely increase activation of the immediate early gene *Fos* in striatal dMSNs and iMSNs (Badiani et al., 1999; Uslaner et al., 2001; Ferguson and Robinson, 2004). *Fos* encodes a number of proteins, including ΔFosB, that have been widely implicated in addiction pathology, and enhanced activation in the NAc is thought to contribute to long-term disruptions in normal C-BG-T activity (Nestler et al., 2001). Co-administration of nicotine, alcohol, or heroin enhances psychostimulant-induced DA release into the NAc (Bunney et al., 2001; Mello et al., 2014; Pattison et al., 2014), though specific combinations do so via divergent mechanisms. For example, administration of cocaine and nicotine simultaneously activates VTA^DA^ neurons and disrupts DA reuptake (Mello et al., 2014; De Moura et al., 2019), resulting in a greater magnitude of DA release into the NAc than that evoked from either drug alone. Notably, some polydrug combinations that include psychostimulants have divergent and opposing effects on the C-BG-T circuit. Co-administration of alcohol with cocaine induces hepatic production of cocaethylene, which can target DAT and presynaptic D_2_ autoreceptors to amplify DA release (Bunney et al., 2001). However, alcohol also prevents cocaine-induced glutamate transmission in the NAc core (Stennett et al., 2020). Lastly, polydrug studies with psychostimulants have identified an exacerbation of drug-induced cellular toxicity compared to psychostimulant use alone. Specifically, co-administration of heroin with cocaine decreases metabolic activity, increases intracellular Ca^2+^ signaling, and decreases mitochondrial membrane potential (Cunha-Oliveira et al., 2008). Collectively, these effects contribute to enhanced caspase 3-dependent apoptotic activity and subsequent cell death compared to either drug alone (Cunha-Oliveira et al., 2008).

#### Nicotine

Unlike psychostimulants, nicotine enhances DA release via activation of nicotinic acetylcholine (nACh) receptors within the VTA. nACh receptors are non-selective cation channels, with permeability to Na^+^, K^+^, and Ca^2+^, and their activation leads to depolarization and enhanced neurotransmitter release (Benowitz, 2009). The reinforcing effects of nicotine are primarily due to nicotine’s activity on somatodendritic nACh receptors located on VTA^DA^ neurons and on presynaptic nACh receptors located on PFC^GLU^ inputs (Nisell et al., 1994). Acute nicotine exposure activates both presynaptic PFC inputs (Fu et al., 2000; Picciotto and Kenny, 2013) and VTA^DA^ cell bodies (Calabresi et al., 1989; Pidoplichko et al., 1997), triggering phasic DA release into the NAc (Dani and De Biasi, 2001; Picciotto and Mineur, 2014). Notably, nicotine-evoked DA release is occluded with blockade of glutamate receptors or activation of GABA_B_ receptors (Fadda et al., 2003; Kosowski et al., 2004), highlighting the extensive regulation of VTA^DA^ neuron activity by local VTA microcircuitry. Studies have not examined how the acute effects of nicotine are changed by polydrug use.

#### Opioids

Although opioids are differentiated by their origin, potency, and receptor bias factor (Schmid et al., 2017), all opioids exert their rewarding effects via activation of mu opioid (μO) receptors. μO receptors are expressed both somatodendritically and axonally (Arvidsson et al., 1995), but the primary mechanism of opioid-induced DA release relies on presynaptic inhibition of VTA^GABA^ neurons. μO receptors are inhibitory G protein-coupled receptors (GPCRs), and their activation reduces neuronal excitability via four mechanisms: (1) Gα-mediated inhibition of cAMP-dependent signaling cascades (e.g. PKA, CREB), (2) Gβγ-mediated activation of G protein-coupled inwardly rectifying K^+^ (GIRK) channels, (3) Gβγ-mediated inactivation of voltage-gated Ca^2+^ channels, and (4) Gβγ-mediated inhibition of SNARE-dependent vesicle release (Bourinet et al., 1996; Blanchet and Lüscher, 2002; Blackmer et al., 2005; Al-Hasani and Bruchas, 2011; Zamponi and Currie, 2013). Acute exposure to opioids inhibits VTA^GABA^ neurons (Johnson and North, 1992; Corre et al., 2018), resulting in subsequent disinhibition of VTA^DA^ neurons and phasic DA release into the NAc (Hemby et al., 1995; Pontieri et al., 1995). Nonetheless, although opioids facilitate DA release into the striatum, whether this DA transmission is necessary for opioid reward remains a point of debate (Badiani et al., 2011). Opioids activate VTA^DA^ neurons *in vivo* and increase DA in the NAc (Di Chiara and Imperato, 1988; Johnson and North, 1992), but neither lesions of the NAc nor systemic antagonism of DA receptor blockades have an effect on opioid reward (Ettenberg et al., 1982; Van Ree and Ramsey, 1987; Olmstead and Franklin, 1997; Sellings and Clarke, 2003). Given the divergent mechanism of action for opioids compared to psychostimulants and nicotine, it is not surprising that co-administration of these drugs augments the acute effects of opioids. Indeed, simultaneous administration of opioids and psychostimulants produces an additive increase in DA release in the NAc, and prolongs elevated levels of DA and its metabolites, DOPAC and HVA (Zernig et al., 1997). Similarly, simultaneous administration of opioids and nicotine enhances opioid-evoked DA release in the NAc and dorsal striatum (Vihavainen et al., 2008). Cross-tolerance to opioid-mediated analgesia has also been shown following pre-exposure to nicotine and cannabis (Schmidt et al., 2001), and chronic nicotine treatment dose-dependently reduces analgesic tolerance to opioids in a nACh receptor-dependent manner (Haghparast et al., 2008; De Moura et al., 2019). Interestingly, pretreatment with Ca^2+^ channel blockers or naloxone prevents this tolerance, suggesting a complex pharmacological interaction between opioids and nicotine (Biala and Weglinska, 2006). Notably, cross-tolerance to opioid analgesia is mediated via divergent mechanisms for different polydrug combinations. Nicotine and opioid cross-tolerance is mediated by μO and nACh receptors (Haghparast et al., 2008; De Moura et al., 2019) while cannabinoid and opioid cross-tolerance is mediated by μO receptors and cannabinoid 1 (CB_1_) receptors (Pugh et al., 1994, 1996).

#### Cannabinoids

Cannabis contains two principal cannabinoids with varying affinity for cannabinoid (CB) receptors: THC is a partial agonist with moderate affinity for both CB_1_ and CB_2_ receptors whereas CBD has extremely low affinity for CB_1_ and CB_2_ receptors and signals through an unknown mechanism (Pertwee, 2008). Interestingly, pretreatment with a range of CBD doses has no effect on THC self-administration (Wakeford et al., 2017), indicating non-overlapping signaling pathways for each cannabinoid. CB_1_ receptors are expressed on presynaptic terminals throughout the CNS and are responsible for the psychoactive effects of cannabis, while CB_2_ receptors are primarily expressed on immune cells of the CNS and PNS and are primarily responsible for the antinociceptive and anti-inflammatory effects of cannabis (Pertwee, 2008). Both subtypes signal through inhibitory GPCR signaling pathways (similar to opioids), and activation of the receptors results in presynaptic inhibition (via activation of GIRK channels and inhibition of VG Ca^2+^ channels) and downregulation of cAMP-dependent signaling cascades. CB_1_ receptors are heavily expressed on presynaptic terminals of VTA^GABA^ neurons as well as NAc dMSNs that target VTA^DA^ neurons (Szabo et al., 2002; Lupica et al., 2004), and activation of CB_1_ receptors reduces GABA-mediated inhibitory postsynaptic currents in VTA slices (Cheer et al., 2000). CB_1_-mediated disinhibition of VTA^DA^ neurons results in an increase in burst firing (French et al., 1997; Diana et al., 1998) and subsequent release of DA into the NAc (Ton et al., 1988; Chen et al., 1990; Fadda et al., 2006), and these effects are blocked by systemic or intra-VTA naloxone (Chen et al., 1990; Tanda et al., 1997). Similar to other drugs, the acute effects of cannabinoids on C-BG-T network activity are broad and engage multiple neurotransmitter systems. For example, acute THC increases both DA and glutamate signaling in the NAc and PFC (Pistis et al., 2002a, b), but decreases GABA signaling in the VTA and PFC (Cheer et al., 2000; Pistis et al., 2002a). Collectively, these alterations in signaling reduce inhibitory feedback within the C-BG-T and facilitate behavioral output. Alcohol consumption prior to cannabis use enhances plasma THC levels and increases self-reported euphoria in humans (Lukas and Orozco, 2001), indicating synergistic effects between the two drugs. Moreover, simultaneous administration of cannabinoids with psychostimulants or opioids enhances activation of VTA^DA^ neurons (Pistis et al., 2004), and simultaneous administration of THC and nicotine increases cFos activation throughout the C-BG-T (Valjent et al., 2002).

#### Alcohol

Despite its widespread use, much less is known about the mechanisms underlying the acute effects of alcohol. Alcohol activates dissociated VTA^DA^ neurons (Brodie et al., 1999) and induces DA release into the NAc (Weiss et al., 1993; Pontieri et al., 1996; Lecca et al., 2006), similar to other potentially addictive drugs. However, GABAA receptors are known to play a central role in the effects of alcohol (Hyytiä and Koob, 1995; Lobo and Harris, 2008). For example, alcohol potentiates GABA_A_ signaling both in cortical slices and neuronal cultures (Aguayo, 1990; Reynolds and Prasad, 1991; Reynolds et al., 1992; Tatebayashi et al., 1998). Given that NAc dMSNs selectively inhibit VTA^GABA^ neurons via GABA_A_-mediated signaling (Edwards et al., 2017), it is possible that alcohol facilitates phasic DA release from VTA^DA^ neurons via inactivation of local VTA^GABA^ neurons. In support of this hypothesis, alcohol inhibits VTA^GABA^ neurons (Steffensen et al., 2009), and intra-VTA infusion of GABA_A_ agonists dose-dependently increase DA release (Kalivas et al., 1990). In addition to GABA, the acute effects of alcohol are dependent on glutamatergic signaling within the C-BG-T (Grant and Colombo, 1993; Krystal et al., 1994). Alcohol increases glutamate release in the NAc and VTA via activation of presynaptic D_1_ receptors (Nie et al., 1994; Xiao et al., 2009), suggesting that alcohol engages a feedforward loop for activation of VTA^DA^ neurons. Polydrug use with alcohol produces synergistic effects throughout the C-BG-T, likely as a result of alcohol’s unique pharmacological profile. For example, chronic pretreatment with nicotine enhances acute alcohol-induced DA release in the NAc (Johnson et al., 1995; Blomqvist et al., 1996), and elevated levels of DA, DOPAC, and HVA persist for over an hour (Tizabi et al., 2002, 2007; Ding et al., 2012). Additionally, alcohol and nicotine co-administration acutely increase production of BDNF and GDNF in the NAc (Truitt et al., 2015), along with increases in glutamatergic signaling in the VTA and PFC (Deehan et al., 2015; Engle et al., 2015). Notably, this wide activation of the C-BG-T network is absent following administration of either drug alone, demonstrating a unique mechanism of action for alcohol and nicotine polydrug use.

### Mechanisms of Addiction: Long-Term Alterations

**FIGURE 5:**
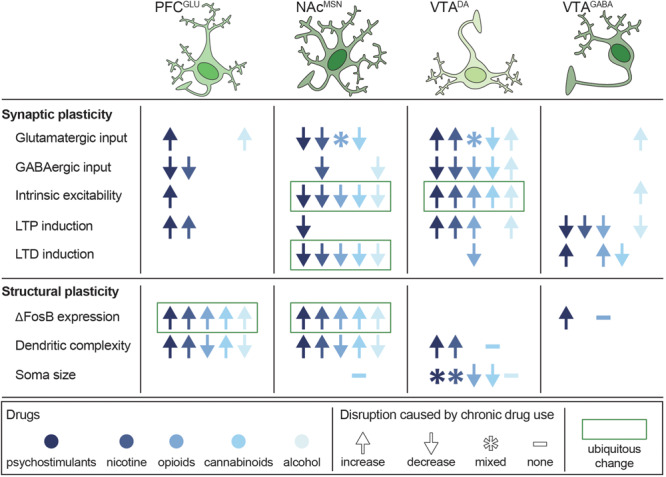
Persistent disruptions in synaptic and structural plasticity caused by long-term use of potentially addictive drugs. Studies are organized by cell type (columns) and type of disruption (rows), with symbols depicting the net change in plasticity and color depicting which drug was tested. Data from Bonci and Williams (1996), Kang et al. (1996, 1998), Robinson and Kolb (1997), Robinson and Kolb (2004), Badiani et al. (1999), Mansvelder and McGehee (2000), Dahchour and De Witte (2000), Uslaner et al. (2001), Brown and Kolb (2001), Robinson et al. (2002), Saal et al. (2003), Amantea et al. (2004), Hamilton and Kolb (2005), Nasif et al. (2005), Kolb et al. (2006), Kolb et al. (2018), Huang et al. (2007), Zhou et al. (2007), Van Den Oever et al. (2008), Kalivas et al. (2009), Niehaus et al. (2010), Russo et al. (2010), Bowers et al. (2010), Spiga et al. (2010), Levine et al. (2011), Dacher and Nugent (2011), Kroener et al. (2012), Lobo et al. (2013), Trifilieff and Martinez (2013), Mello et al. (2014), Peterson et al. (2015), Bloomfield et al. (2016), Creed et al. (2016), Ehlinger et al. (2016), Hearing et al. (2016), Morud et al. (2016), Friend L. et al., 2017), Langlois and Nugent (2017), Edwards et al. (2017), Spencer et al. (2018), You et al. (2018), Hwang and Lupica (2019), Kruse et al. (2019), McDevitt et al. (2019), Neuhofer et al. (2019), Pickel et al. (2019), and Ponzoni et al. (2019).

Long-term use of psychostimulants, nicotine, opioids, cannabinoids, and alcohol results in widespread and disparate changes throughout the C-BG-T network, yet there are notable alterations that are shared across drugs (Figure 5). These long-term adaptations contribute to transitions from binge/intoxication phases to withdrawal and negative affect, followed by preoccupation and compulsive drug-seeking (Koob and Volkow, 2016). For example, acute withdrawal produces a transient reduction in tonic DA levels in the NAc (Weiss et al., 1992; Diana et al., 1993, 1998; Hildebrand et al., 1998). This is followed by a persistent increase in excitability of VTA^DA^ neurons (Mansvelder and McGehee, 2000; Bloomfield et al., 2016; Creed et al., 2016; Langlois and Nugent, 2017; You et al., 2018), which contributes to enhanced cue-evoked phasic DA release during abstinence (Volkow et al., 2011). Conversely, withdrawal also produces a persistent reduction in long-term depression (LTD) and intrinsic excitability in NAc MSNs, as well as a reduction in striatal D_2_ receptor binding (Trifilieff and Martinez, 2013). As described earlier, striatal D_2_ receptors are primarily expressed on iMSNs, can serve as a “stop” signal on the C-BG-T circuit, and reduced D_2_ availability has been ubiquitously linked to a range of addictive diseases, including drug addiction and obesity (Volkow and Morales, 2015; Kravitz et al., 2016; Friend D.M. et al., 2017). Finally, these drugs all drive a persistent increase in ΔFosB expression in cortical pyramidal cells and NAc dMSNs (Lobo et al., 2013), which has been linked to drug-seeking during abstinence (Nestler et al., 2001). Importantly, although chronic use of any of these drugs results in a multitude of other transient and/or persistent changes across the C-BG-T network, it is beyond the scope of the current review to provide an exhaustive summary. Rather, the focus will be on changes in plasticity, morphology, and connectivity within the VTA, NAc, and PFC following both single and polydrug use, with an emphasis on how the antagonistic and synergistic effects of these drugs can differentially disrupt C-BG-T network dynamics.

#### Psychostimulants

Psychostimulants produce long-term disruptions in glutamate homeostasis and alterations in neuronal morphology throughout the C-BG-T network (Kalivas, 2009; Badiani et al., 2011). Repeated cocaine administration weakens GABA_A_-mediated inhibition of prelimbic (PrL) pyramidal neurons, increasing their excitability and augmenting excitatory drive to the NAc (Nasif et al., 2005; Huang et al., 2007). Similarly, chronic cocaine increases glutamatergic input to the VTA (Saal et al., 2003; Bowers et al., 2010) and weakens GABA_B_-mediated inhibition of VTA^DA^ neurons (Bonci and Williams, 1996; Edwards et al., 2017), leading to a facilitation of VTA^DA^ neuron activity. Additionally, psychostimulants generate silent synapses on dMSNs via synaptogenesis (Boudreau et al., 2007; Graziane et al., 2016) and depress glutamate release from PrL inputs to the NAc core (Bamford and Wang, 2019), weakening striatal output. Importantly, cocaine-silenced synapses on dMSNs can be unsilenced during withdrawal via recruitment of AMPA receptors (Boudreau et al., 2007; Graziane et al., 2016), and a low dose psychostimulant challenge restores glutamate release into the NAc core (Boudreau et al., 2007; Bamford and Wang, 2019), suggesting the promotion of allostasis. Finally, repeated psychostimulant exposure increases expression of ΔFosB in PFC pyramidal neurons and NAc dMSNs (Perrotti et al., 2005), which contributes to an increase in dendritic branching that persists for at least 30 days of abstinence (Robinson and Kolb, 1997, 2004; Russo et al., 2010). Studies have not examined how these effects of psychostimulants are changed by polydrug use.

#### Nicotine

Similar to psychostimulants, long-term nicotine administration reduces GABA_B_-mediated signaling in the mPFC and NAc (Amantea et al., 2004), dampening inhibitory drive onto the C- BG-T network. Additionally, nicotine enhances glutamatergic input to VTA^DA^ neurons (Mansvelder and McGehee, 2000; Saal et al., 2003), and sensitizes evoked DA release into the NAc (Benwell et al., 1995). Repeated nicotine use also leads to an upregulation of nicotinic acetylcholine receptors throughout the C-BG-T, including the PFC and midbrain (Marks et al., 1983; Benwell et al., 1988; Breese et al., 1997). Conversely, chronic nicotine exposure weakens both glutamatergic and GABAergic inputs to the NAc during abstinence, potentially via enhanced sensitivity of NAc D_2_ receptors (Morud et al., 2016). Because NAc output is heavily regulated via local GABAergic microcircuitry, increased activity of iMSNs would likely dampen overall NAc output. This is supported by the upregulation of Ca^2+^-permeable AMPA receptors in the NAc that are capable of conducting Ca^2+^ in the absence of NMDA receptor activation (Ponzoni et al., 2019). Long-term nicotine use also produces a persistent increase in mPFC pyramidal cell and NAc MSN dendritic branching (Brown and Kolb, 2001; Hamilton and Kolb, 2005; Ehlinger et al., 2016), similar to psychostimulants. Moreover, adolescent nicotine use enhances synaptic pruning, microglial activation, and inflammatory cytokine expression throughout the C-BG-T via a D_2_ receptor-mediated mechanism (Linker et al., 2020). Repeated co-administration of psychostimulants and nicotine augments long-term potentiation (LTP) induction and ΔFosB in the NAc core (Levine et al., 2011; Mello et al., 2014). Interestingly, these effects are absent following sequential administration of these drugs (Levine et al., 2011), indicating a unique pharmacological profile for concurrent nicotine and psychostimulant administration.

#### Opioids

Similar to psychostimulants and nicotine, long-term exposure to opioids strengthens glutamatergic input to VTA^DA^ neurons (Bonci and Williams, 1996; Saal et al., 2003). They also weaken GABAergic inhibition of VTA^DA^ neurons (Mansvelder and McGehee, 2000; Niehaus et al., 2010; Dacher and Nugent, 2011) via a reduction of dMSN-mediated GABA_B_ inhibition (Bonci and Williams, 1996). This effect is largely driven by MSNs in the NAc (Yang et al., 2018), indicating a persistent disruption in NAc output. Unlike psychostimulants and nicotine, however, long-term exposure to opioids decreases dendritic branching and spine density in NAc MSNs and mPFC pyramidal cells (Badiani et al., 1999; Robinson et al., 2002; Van Den Oever et al., 2008). Within the NAc, long-term opioid exposure weakens glutamatergic input to NAc shell iMSNs (Hearing et al., 2016; McDevitt et al., 2019) and induces silent synapses on iMSNs via AMPA internalization (Graziane et al., 2016). Moreover, during withdrawal, opioid-generated silent synapses on iMSNs are eliminated (Graziane et al., 2016), and the intrinsic excitability of iMSNs is weakened (McDevitt et al., 2019). Given that MSNs create a dense network of lateral inhibition within the NAc, with ∼30% of iMSNs synapsing onto other iMSNs or dMSNs (Taverna et al., 2008), it is possible that opioid-induced disruptions in iMSN signaling could disrupt local NAc microcircuitry and facilitate aberrant C-BG-T network dynamics. Studies have not examined how these effects of opioids are changed by polydrug use.

#### Cannabinoids

Repeated THC administration weakens glutamatergic signaling from the mPFC to the NAc (Spencer et al., 2018; Hwang and Lupica, 2019; Neuhofer et al., 2019) but strengthens input from the basolateral amygdala and ventral hippocampus to the NAc shell (Hwang and Lupica, 2019), suggesting a rewiring of excitatory input to the NAc following long-term cannabinoid exposure. Notably, repeated THC administration also occludes CB_1_-mediated LTD on VTA^GABA^ neurons (Friend L. et al., 2017), indicating a loss of local inhibitory drive on VTA^DA^ neurons. Additionally, adolescent THC administration followed by abstinence reduces intrinsic excitability of PrL neurons (Pickel et al., 2019) and weakens glutamatergic input to the VTA (Kruse et al., 2019), indicating the presence of a hypoglutamatergic state induced by cannabinoids. THC exposure also produces long-lasting changes in morphology throughout the C-BG-T, increasing dendritic spine length and branching in the mPFC and NAc shell (Kolb et al., 2006) and dendritic spine density in the NAc shell (Kolb et al., 2018), but reducing the size of VTA^DA^ neurons (Spiga et al., 2010; Behan et al., 2012). Repeated co-administration of THC and nicotine enhances expression of ΔFosB in the NAc, and acute withdrawal dysregulates glutamatergic input to the NAc and PFC (Ponzoni et al., 2019).

#### Alcohol

Similar to opioids, extended alcohol exposure decreases dendritic branching and spine density in the NAc shell and mPFC (Zhou et al., 2007; Peterson et al., 2015), and withdrawal from alcohol reduces tonic DA levels in the NAc (Rossetti et al., 1992; Diana et al., 1993). However, alcohol withdrawal also causes distinct changes throughout the C-BG-T network, including reduced GABAergic signaling in the NAc and hippocampus (Kang et al., 1996, 1998; Dahchour and De Witte, 2000) and increased glutamatergic signaling in the NAc and PFC (Dahchour and De Witte, 2000; Kroener et al., 2012). Notably, this increased glutamatergic signaling is due to disrupted glutamate reuptake, rather than enhanced glutamate release (Pati et al., 2016). *In vitro* polydrug exposure to alcohol and nicotine induces a 2.5-fold increase in caspase-3 activation, elevating apoptotic cascades and driving cell death (Ramlochansingh et al., 2011). Interestingly, however, alcohol or nicotine withdrawal-induced neurodegeneration is less severe following co-administration of both drugs (Oliveira-da-Silva et al., 2010), indicating a unique molecular pathology following nicotine and alcohol polydrug use.

## Conclusion

Improving the translatability and mapping of behavioral measures in preclinical models to accurately reflect polysubstance history and dependency in clinical populations is essential. This is particularly true, as human imaging studies are limited by an inability to control for intake history, making behavioral models in other species advantageous for assessing polydrug history under controlled intake conditions. However, these models are limited in their capacity to fully encompass the complex social and environmental contexts that contribute to the unique use patterns for multiple substances with addiction potential. Nevertheless, when designing experiments in clinical or preclinical populations, factors such as time of day of intake, temporal proximity of intake, and environmental preferences for administration must be consideredfor each substance class. For instance, cocaine use is predominantly favored outside of home environments, whereas heroin use is greater in “home” contexts in both humans and rodents (Caprioli et al., 2009; Badiani and Spagnolo, 2013; De Pirro et al., 2018; De Luca et al., 2019). In addition, route of drug administration (e.g. oral ingestion, injection, and inhalation) is especially important to factor into studies, as it leads to unique patterns of polysubstance history that may impact the developmentand severity of addiction behaviors (Roy et al., 2013).

Accounting for temporal patterns of drug use is also essential. Although many studies have focused on simultaneous drug combinations, sequential patterns of polydrug consumption are more frequently reported (Leri et al., 2004; Roy et al., 2013) and produce unique circuit adaptations following acute and repeated drug exposure (Cunha-Oliveira et al., 2008). Understanding sex differences in frequency and pattern of polydrug use (McClure et al., 2017), drug discrimination (Spence et al., 2016), and circuit alterations (Canterberry et al., 2016; Manwell et al., 2019) is also necessary to fully understand the interactions and impacts of polydrug use in clinical populations. Furthermore, although powerful behavioral economic models allow comparisons across drug classes, these experiments must be designed with consideration of different scales of intake and indifference points for drug valuation in order to accurately model parameters and interpret data (Newman and Ferrario, 2019). As these factors, coupled with the interplay of socioeconomic background, social environment, and access strategies, affect frequency and susceptibility to polydrug use (Gjersing et al., 2013; Hernández-Serrano et al., 2018), encompassing these influences in preclinical models is crucial for translational relevance of findings.

It is important to appreciate that novel preclinical paradigms are continuously being developed to model different routes of drug administration and to study relapse under clinically relevant conditions. For instance, recently established models allow for voluntary control over self-administration of vaporized ethanol (de Guglielmo et al., 2017; Kimbrough et al., 2017), cannabis (McLaughlin, 2018; Freels et al., 2020), and nicotine (Marusich et al., 2019). In addition, multiple models for inducing drug abstinence are being introduced. For instance, voluntary abstinence is achieved via pairing of drug-taking with adverse consequences such as shock-lever pairings or electrified barriers placed in front of levers (Krasnova et al., 2014; Venniro et al., 2016), andthrough choice procedures involving a drug versus an alternative reinforcer (e.g. palatable food or social interaction) (Venniro et al., 2016, 2017, 2019). These paradigms are notable for their high translational value and may be powerful for understanding the neural basis of therapies such as contingency management, in which abstinence from drug use results in a monetary reward. Combining these models with behavioral economic paradigms could clarify how the relative value of drugs and alternative reinforcers changes with polydrug use to improve treatment efficacy. Addiction is a complex disease with multiple, highly variable factors contributing to the initiation and maintenance of drug use, as well as relapse. Given the widespread prevalence of polydrug use among drug users, it is critical that we incorporate this variablility into human studies and animal models. This will help to determine if polysubstance use exacerbates SUD severity, if increased SUD severity drives polysubstance use, or if there is a bidirectional relationship between the two. Though challenging, understanding the behavioral, genetic, and environmental contributions to polysubstance use and addiction, as well as the mechanisms that underlie addiction-severity and relapse, will aid in developing efficacious treatment and policy strategies to combat this ongoing public health crisis.

## Author Contributions

All authors wrote and revised the manuscript, as well as read and approved the submitted version.

## Conflict of Interest

The authors declare that the research was conducted in the absence of any commercial or financial relationships that could be construed as a potential conflict of interest.
